# The Role of Expert Judgment in Statistical Inference and Evidence-Based Decision-Making

**DOI:** 10.1080/00031305.2018.1529623

**Published:** 2019-03-20

**Authors:** Naomi C. Brownstein, Thomas A. Louis, Anthony O’Hagan, Jane Pendergast

**Affiliations:** a Department of Biostatistics and Bioinformatics, Moffitt Cancer Center, Tampa, FL;; b Department of Oncologic Sciences, University of South Florida, Tampa, FL;; c Department of Behavioral Sciences and Social Medicine, Florida State University, Tallahassee, FL;; d Department of Biostatistics, Johns Hopkins Bloomberg School of Public Health, Baltimore, MD;; e School of Mathematics and Statistics, The University of Sheffield, Sheffield, UK;; f Department of Biostatistics and Bioinformatics, Duke University, Durham, NC

**Keywords:** Bayesian paradigm, Clinical trials, Collaboration, Elicitation, Scientific method, Subjectivity

## Abstract

This article resulted from our participation in the session on the “role of expert opinion and judgment in statistical inference” at the October 2017 ASA Symposium on Statistical Inference. We present a strong, unified statement on roles of expert judgment in statistics with processes for obtaining input, whether from a Bayesian or frequentist perspective. Topics include the role of subjectivity in the cycle of scientific inference and decisions, followed by a clinical trial and a greenhouse gas emissions case study that illustrate the role of judgments and the importance of basing them on objective information and a comprehensive uncertainty assessment. We close with a call for increased proactivity and involvement of statisticians in study conceptualization, design, conduct, analysis, and communication.

## 1 Introduction

As participants in the October 2017 Symposium on Statistical Inference (SSI), organized and sponsored by the American Statistical Association (ASA), we were challenged to host a session and write a paper inspired by the question, “Do expert opinion and judgment have a role in statistical inference and evidence-based decision-making?” While we work from different perspectives and in different statistical paradigms (both frequentist and Bayesian), there was a resounding “yes!” among us, with ample common ground in our thinking related to this infrequently discussed and often under-appreciated component of statistical and scientific practice.

Expert judgment is a feature of both frequentist and Bayesian inference. Judgments are subjective, and subjectivity is a topic that has generated heated debate in the Statistical community (Gelman and Hennig 2017). The subjectivity in a Bayesian prior distribution has been criticized by frequentists. Implicit claims are that frequentist methods are objective and that they, “let the data speak for themselves.” However, frequentist methods also require some components of subjectivity in the choice of a model and of estimators, test statistics, etc. These choices are subjective in the sense that every expert builds knowledge and judgment into their own personal framework of understanding, which is not likely to be identical to that of anyone else. Expert judgment is clearly needed for valid statistical and scientific analyses. Yet, questions regarding how, when, how often, and from whom judgment is helpful, rather than leading to biased, misleading and nonreproducible results, is less clear.

The ASA commissioned this special issue of *The American Statistician* to stimulate “a major rethinking of statistical inference, aiming to initiate a process that ultimately moves statistical-science—and science itself—into a new age.” We consider the roles of expert judgment in scientific practice. This article is a distillation of our common ground, along with our advice, warnings, and suggestions for incorporating expert judgment into science, while maintaining the integrity and scientific rigor of the research. Note that although we were asked in the Symposium to consider expert opinion and judgment, we avoid the word “opinion” because it risks being equated to uninformed rhetoric. We emphasize “judgment” that should be informed, carefully considered and transparent. While we might still disagree on specific details or the relative importance of various points, we present a strong, unified statement related to this key component of statistical practice. Additional literature on expert judgment in statistics may be found in a companion paper (Brownstein 2018)

Our article is organized in the following sections where we share our thoughts on when and how expert judgment has a legitimate and necessary role in scientific inquiry. Section 2 presents the role of judgment in the scientific method and more generally, in four stages of scientific inquiry, inference, and decision-making. In Section 3, the four stages are examined in more detail, focusing on their needs for expert judgment and the qualifications implied by “expert.” Two case studies are presented in Section 4, with emphasis on the principled and scientific application of expert judgments. Finally, Section 5 summarizes our key conclusions.

## 2 The Cycles of Inference and Decision in Science

The practice of science is often described in terms of the scientific method, defined in Oxford University Press (2018d) as involving, “systematic observation, measurement, and experiment, and the formulation, testing, and modification of hypotheses.” More generally, one might describe the scientific method as a collection of methodologies and processes of gathering observable, measurable evidence using experimentation and careful observation, and of analyzing the evidence to draw conclusions, inform decisions and raise new questions. The purpose may be to inform about pathways or mechanisms by which results are obtained or to aid in prediction or estimation of quantities of interest. A thorough review of the history of the scientific method is found in Anderson and Hepburn (2016). While we believe that not all scientific inquiry, and certainly not all decision-making, falls within that prototypical guide, it is important to understand how the scientific method fits into our framework. Namely, science is data-driven in the ascertainment of objective information to draw scientific conclusions, yet with expert judgment feeding into the processes.

**Fig. 1:**
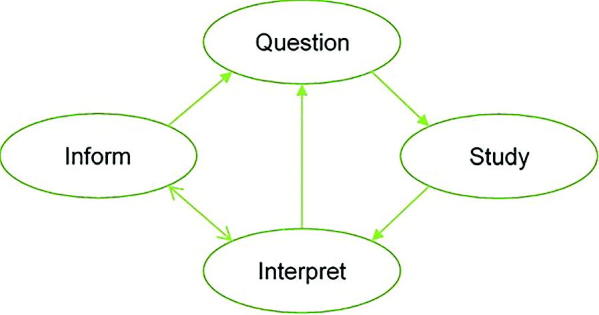
The cycles of inference and decision.

We base our four-stage graphic (Figure 1) on the depiction of Garland (2016), adding the Inform stage to accommodate decision-making and broadening the definitions of the other stages. It illustrates four stages in the perpetual process of scientific inquiry and evidence-based decision-making: Question, Study, Interpret, and Inform. These stages are formulated in highly general terms, because the practice of scientific inquiry, and hence the remit of statistical analysis, is also very wide.

We first describe activities that comprise each of these stages. Our framework allows us to underscore the varied roles of expert judgment throughout the perpetual cycles of the scientific method.

1. *Question*: Scientific inquiry can be characterized as beginning with one or more questions. A Question might be a formal scientific hypothesis arising either out of observation of real-world phenomena or from the current status of scientific knowledge and debate. On the other hand, a Question could be posed outside the scientific community, such as a request for evidence-based input to inform an impending policy decision. It might also simply express a wish to estimate more accurately certain quantities or parameters in current scientific theories.
2. *Study*: To address the Question scientifically, it is necessary to gather evidence in a rigorous manner. In the Study stage, we include all aspects of study design, including design of observational studies, experiments, questionnaires, systematic literature reviews, and meta-analyses. The Study may also involve sequential design or the design of a number of distinct, possibly concurrent studies. We also include in this stage the conduct of the study, resulting in some form of data.
3. *Interpret*: In the Interpret stage, data resulting from the Study are employed to address the Question. Typically, this may involve descriptive statistics and statistical inference, such as parameter estimation and hypothesis testing. In a Bayesian analysis, the primary statistical result might simply take the form of a posterior distribution. However, the “Interpret” stage should also embed the findings of the analysis into the wider body of science to which it refers, thereby updating that body of knowledge. In doing so, the wider implications of the findings will emerge.
4. *Inform*: The Interpret stage will often suggest new Questions, and a new cycle of scientific investigation will thereby be initiated. First, however, the Interpret stage usually will be followed by the Inform stage. For a formal scientific study, findings should be formally written and communicated in peer-reviewed outlets, such as conferences, journals, and books. In fact, peer-review may lead to revisions in the Interpret stage before formal publication of the findings. Subsequent examination and evaluation of published studies by the scientific community may in turn lead to new interpretations of existing studies and a new Question, leading to a new Study. Where the Question is a request for input to a decision, the Inform stage is when the results of the Study are communicated to facilitate the decision-making process. New Questions may arise based on the output produced in the Inform stage. Alternatively, the original Question may need to be revisited in one or more future Studies, especially when the evidence is not yet adequate to merit a robust conclusion.

## 3 Science and Subjectivity

In all stages of the scientific method defined in Section 2, expert judgment and knowledge are required. The relevant definitions are first presented. First, knowledge is defined (Oxford University Press 2018b) as, “facts, information, and skills acquired by a person through experience or education; the theoretical or practical understanding of a subject.” Judgment is defined (Oxford University Press 2018a) as, “the ability to make considered decisions or come to sensible conclusions.” By contrast, opinion (Oxford University Press 2018c) is, “a view or judgment formed about something, not necessarily based on fact or knowledge.”

We consider that judgment implies using information and knowledge to form an assessment or evaluation. While it is possible that opinions can be similarly well-informed, by definition, opinion does not necessarily imply that external information was incorporated into the evaluation. It may simply be a view, judgment, or appraisal that fits within a belief system or is comfortable for some other reason. While “judgment” is normally thought of as being based on observable truth, “opinion” can be simply based on preference. The subjectivity in this article refers to judgment of experts based on knowledge, skill, and experience. We examine the roles of expert judgment in each of the four stages presented in Figure 1, paying particular attention to integration of statistical and *content* expertise. We use content expertise to refer to the discipline expertise in which the Question arises. For more detail on expert scientific judgment, please see Brownstein (2018).

### 3.1 The Question Stage

When developing the Question, we rely heavily on the expert judgment of the content experts. The Question may arise from identification of a barrier or problem in need of an answer, a quest to understand the “why” behind some phenomenon, event, or process, or simply to better quantify some parameter, effect or disturbance. For example, one might ask “Why are some people able to fend off the negative impacts of an HIV infection while others cannot?” Knowledge of the literature and what other experiments or studies others have done to address this Question or related Questions is critical. Where the Question arises from a request for scientific input to inform a decision, the content experts serve key roles in formulating specific questions, such as “What can we say about the toxicity of this pollutant for fish in European rivers?”, or “Which areas in this catchment will be flooded if the catchment experiences a once-in-100-years weekly rainfall?”

While the content experts have primary responsibility to develop the Question, statisticians can elicit clarity on the framing of the Question by asking pertinent questions from their perspective. Inquiry from the statistician serves not only to establish and confirm the statistician’s understanding of the Question and its scientific context, but also to translate the question to a statistical framework, which may guide analytic decisions in the Study Stage. Indeed, strong listening and communication skills are critical for both the content and statistical experts! Barry Nussbaum, 2017 President of the ASA, shared his mantra, “It’s not what you said. It’s not what they heard. It’s what they say they heard” (Nussbaum 2017). Communication can be improved by echoing back your understanding of how you interpreted what you heard, and asking others to echo their understanding of the points you have made. Producing a written summary of the Question, evidence-based justification for the importance of the Question, and evidence needed to answer the Question allows the research team members a chance to check on how well they are communicating and where points of disagreement may still exist.

If the Question is seeking information on potential pathways or mechanisms, evidence for or against competing theories is presented, and decisions must be made on the rationale for how the Question will be pursued. The content experts play the main role here, but statistical expertise can be helpful when framing the Question to bring up potential statistical issues with the proposed approach.

Part of defining the Question is determining what evidence, measures and parameters would be useful and adequate to arrive at an answer. Properties of those measurements, including validity, reliability, cost, and distributional properties are considered. This is an area in which both content and statistical experts can contribute. For example, content experts may be focused on what data would be needed and whether primary data collection would be needed or existing secondary data would suffice. If the study needs to collect primary data, there will be a need for discussion of exactly what information will be desired and how to elicit that information. In turn, the statistician will seek to better understand properties of the desired measurements. Moreover, the statistician may highlight unmeasured influences, the impact of missing data, and whether sources of bias, confounding, or variability could be reduced or eliminated by appropriate study design or data collection methods.

In addition, a Study may involve more than one Question. In this case, discussions are needed regarding which Questions are considered primary or secondary, the interrelatedness or independence of the Questions, and whether any of the Questions can be addressed jointly. Statistical and content experts should collaborate in making these decisions.

Thinking through the issues that can (and will!) arise when defining the Question and information needed to address it requires effective collaboration and team effort. Effective collaboration requires mutual respect for team members and personal authenticity; an understanding of the strengths and skill sets of each member; an understanding and buy-in for a common goal; a willingness not only to hear and understand what another has said, but to embrace ideas different from yours; a willingness to ask for evidence and assumptions behind a statement of fact or belief; reliability and consistency in thought and behavior; a willingness to offer alternative ideas or approaches; a willingness to compromise, when appropriate. All of this rely on fluency, both written and oral, in the language used, including technical and discipline-specific terms. Cognizance of acronyms and language shortcuts is the first step to reducing them to enhance communication. We recommend that researchers create a written summary of decisions made in such discussions, and make sure such documents are accessible electronically to all study members for review and editing.

### 3.2 The Study Stage

Once the Question and pertinent measures are defined, the approach to gathering information (data) must be developed. Can the Question be answered in one study, or will it take a series of planned studies? What studies have been done before? Could any component of prior studies be replicated with modification or improvement in this study? Did previous studies report unanticipated problems that could be avoided in this study?

Much of the work in planning a study involves a collaborative effort among all members of the research team. Those researchers who will be “on the ground” collecting information will have expertise on what is feasible and what is not. The statistician can offer their expertise and judgment on many aspects of study design and implementation, such as strengths and weaknesses of different study designs, questionnaire development, psychometric properties of data collection instruments, issues surrounding sources and control of error, replication, operational randomization, and blinding. The content experts will share their knowledge and judgments on the target scope of inference, such as whether to estimate the 100-year flood plane for a large geographic area or just one river; for all people with a disease or only those in a local area who meet defined inclusion criteria. Content experts bring to the discussion additional information that can be considered in the study design, perhaps stemming from theorized or understood pathways, mechanisms, or concurrent influences by which observed outcomes can differ.

If working within the Bayesian framework, statisticians help elicit information from the content experts to feed into the prior distribution. Those approaching the problem from a frequentist perspective will also be looking to prior studies and expert judgment when developing a study design. No matter what analysis approach is used, the ultimate goal remains the same: to collect enough high quality information to effectively address the Question. Here, the word “quality” can encompass many aspects, including reduced variability of measures and removal or control of sources of biases, along with proper data collection and maintenance systems that protect the integrity of the data.

During the Study stage, prior to data collection, there should be enough information to create a study protocol, data monitoring plan, and a statistical analysis plan (SAP), upon which everyone agrees (Finfer and Bellomo 2009; Ellenberg, Fleming, and DeMets 2003; Ott 1991). Such SAPs are becoming more common and recognized by funders as an important part of the research process. While not every study will require these formal documents, the idea is that there is a common understanding on how the study will be conducted; how the data will be collected, monitored, and maintained; and the analytic approach used to address the Question. With the goal of transparency of the body of work to be accomplished, good documentation and data provenance are important components of scientific inquiry.

Unfortunately, forms of scientific malpractice, such as repeating the analysis with a slightly altered question in an attempt to achieve statistically significant results—a practice called *HARKing* (hypothesizing after the results are known)—or testing for many associations without prior hypotheses (*p*-hacking) are commonplace in the scientific literature (Kerr 1998; Head et al. 2015). A well thought-out study design and SAP can help safeguard against urges to reanalyze the data later after obtaining disappointing results. However, it should be noted that the data monitoring process itself involves judgment, as exemplified in Section 4.1.3 and discussed further elsewhere (Pocock 2006).

A related practice of publishing a Registered Report, in which methods and proposed analyses are preregistered and peer-reviewed prior to conducting the research, is gaining momentum. The intent is to eliminate questionable research practices, such as selective reporting of findings and publication bias, by provisional acceptance for publication before data collection begins. Currently, the Center for Open Science lists 121 journals that use the Registered Reports publishing format as an option in regular submissions or as part of special issues (Center for Open Science 2018).

Often, before a study can begin, funding must be obtained. When there are commissioned studies, often researchers need to write a formal proposal to seek funding. A grant proposal provides an opportunity to present not only the justification for the study but also detail on the study process, analysis plan, and how the desired information will address the Question. The grant review process brings in a new external set of experts with judgments of their own on the merits of the proposal. Feedback for a proposal can identify strengths and weaknesses of the proposed work. Reviewers’ expert judgments on whether a particular study should be funded are intended to weed out studies without strong support and justification for both the importance of the Question and the development of the Study.

As the study progresses, important discussions and decisions are likely to be made at research team meetings. It is important that statisticians, like other key members of the team, are present at the table to listen, question, offer insight and expertise, and retain knowledge of decisions made. Written summaries of research meetings, documentation of any decisions made that were not anticipated in the Question or (earlier in the) Study stages or are necessary to successfully implement the study protocol, all promote transparency and reproducibility. Additional documentation for the Study stage that should be available for all team members to view may include computer code management systems, clearly documented analytic code, well-considered file structures and naming conventions, and common file space for all members to see such documentation. Subsequently, the team will synthesize information from the Study stage for use in the Interpret and Inform stages.

### 3.3 The Interpret Stage

The methodology used to analyze the data and the specifications of the assumed model feed into the interpretation of the data. Some analytic methods produce estimated probabilities that relate directly to the Question; others may provide information that helps address the Question, but perhaps more indirectly. The chosen methodology may produce parameter estimates that need to be understood in context of the model, and the alignment of the model to the observed data and prior beliefs needs to be considered.

The expertise of the statistician is needed both to understand the nuances of proper interpretation of the analytic results in context of the executed study, assumptions made, and modeling used and to guard against overinterpretation. For example, a statistician may help protect the team from common misconceptions and malpractice, such as tendencies to extend inferences to populations outside those under study or to interpret association as causation, or failure to consider the impact of unmeasured confounders, mediators or moderators in the interpretation of results. The statistical expert can protect against improper use or interpretation of a *p*-value, discuss the difference between clinical and statistical significance, and highlight the potential impact of missingness mechanisms and violations of statistical and causal assumptions on the results. If working in a Bayesian framework, the statistician can also discuss the impact of potential expertise bias and over or underprecision in specification of the prior.

Similarly, it is not appropriate for statisticians to focus solely on analytic methods and numeric results, or for content experts to delay involving statisticians until the Interpret stage. By not being involved in other study aspects and discussions, the statistician is poorly positioned to make modeling choices, recognize possible biases, and interpret findings in the context of the study as conducted, which could differ from the study as planned.

As the analytic results are interpreted in the framework of the Question and the Study protocol, both the content and statistical experts help the study team blend the (properly interpreted) new findings into their existing knowledge and understanding. This process will likely include team discussions of the clinical meaning and impact these findings might have in the population under study, and how the results could be explained or interpreted within the current framework of understanding. The discussion will include the strength of the evidence, based on posterior distributions, point estimates of key parameters, confidence or credibility intervals, etc. and consistency with prior studies, as well as potential weaknesses or caveats of the study posed by both the statistical and content experts.

### 3.4 The Inform Stage

Once the analytic results have been interpreted within the framework of the study design and measures used to address the Question, it is time to assess what was learned and share that information more broadly. Of course, those who developed the Question and often those who funded the study will need and expect a complete summary of the work done, describing how the results have informed the Question. Indeed, there may be interest in the work outside of academia, such as patients curious about new therapies for their conditions, policy-makers seeking to understand actions that may yield societal benefit, and others simply wondering about current topics and trends in various fields of science.

Scientific reports or publications, where the process, methods, results, and conclusions of a study can be shared broadly, are important tangible outcomes of the Inform stage. In each Inform stage, the team members build on what they knew previously and share what they learned from the findings, whether or not the results obtained were anticipated or exciting. To guard against publication bias, null results, in particular, must be communicated, despite disincentives for doing so (Franco, Malhotra, and Simonovits 2014; Easterbrook et al. 1991).

Strong communication and documentation skills in the Inform stage are paramount to ensure that all components of a study are documented as the study progresses and are presented clearly, completely, and with as much transparency as possible. While the statistician is frequently tasked with managing the sections describing the quantitative methods and results, they should also collaborate throughout the report to provide their input to interpretation and implications of the findings (see Section 3.3). Additional documentation from the Study stage, such as clearly written and readable computer code, can also be included as supplementary material.

The process of creating a scientific manuscript and undergoing the peer-review process for publication is another place in which statistical and content expert judgment enters into both the Interpret and Inform stages. Comments from others on drafts of the manuscript can lead to revised interpretation in light of new information or perspective from that feedback before submission of the manuscript for publication. Once submitted, additional expertise is gathered from the peer reviewers, such as calls for clearer evidence of claims or interpretations made, challenges made to stated justifications of assumptions or interpretations, call for greater transparency or detail in information presented, or citations to related work which may blend into the discussion section. Reviewer comments can greatly impact the quality and clarity of information presented in the final publication.

Recently, journal editors are considering re-examining their review processes, focusing acceptance or rejection on strength of evidence, not *p*-values. Locascio (2017b) argues for a results-blind review system in which the reviewer makes a preliminary decision on the strength of the manuscript without knowing the results. Final decisions would not allow rejection on the basis of *p*-values. Commentary on the feasibility of this approach may be found elsewhere (Marks 2017; Grice 2017; Hyman 2017; Locascio 2017a). We encourage journal reviewers to examine the appropriateness of the methods for the study under consideration, rather than accepting justification of methods simply based on their publication and use elsewhere.

When the Question has arisen to facilitate decision-making, the primary purpose of the Inform stage is to convey the scientific evidence to the decision-maker, after it has been assembled and analyzed in the Study and Interpret stages. Here, too, communication skills are particularly important. Governments are increasingly but not exclusively reliant on evidence for policy- and decision-making (Oliver and de Vocht 2017; HM Treasury 2015; Oliver, Lorenc, and Innvaer 2014; LaVange 2014), and there is much current interest in the challenges of communicating uncertainty to decision-makers (Cairney and Oliver 2017; National Academies of Sciences, Engineering, and Medicine et al. 2017; National Research Council et al. 2012a,b). Despite these challenges, it is essential to measure uncertainty and important to try to communicate it as effectively as possible. Understanding the perspectives of decision-makers (i.e., priorities and goals, options under consideration, risks/benefits), what processes they must follow, and time-constraints are helpful in such communications.

As depicted in our graphic of the scientific method (Figure 1), learning and discovery is cyclical. Once we address one Question, new Questions often arise. Researchers may be interested in the work of other authors in similar fields, perhaps using information from published studies to inform their next study. Sometimes the results obtained are not definitive, or not adequate for robust decision-making, and ways to redirect the next investigation of the same or a revised version of the Question are planned. Other times, a replication Study is conducted based on the same Question simply to see if the results remain qualitatively similar, despite inevitable lack of perfect duplicability in all aspects of the study environment (Lindsay and Ehrenberg 1993).

### 3.5 But Is It Science?

When expert judgments are used in any stage of a scientific inquiry, the outcomes contain subjective elements. The inescapable conclusion is that science itself has a subjective component, aspects of which can be communicated probabilistically, and should be interpreted according to the theory of subjective probability (Anscombe and Aumann 1963). That is, while there may be objective information on which probabilities are based, there also will always be between-individual (scientist) variation.

There can be heated opposition to the notion of subjective science, with objectivity promoted as fundamental to the scientific method, and subjectivity considered as anathema to a true scientist. According to this viewpoint, subjectivity is unscientific, biased, possibly prejudiced. But, science cannot be totally objective. Scientists propose new or amended theories, choose experimental designs or statistical analysis techniques, interpret data, and so on. Although these judgments are subjective, expert judgments result from a synthesis of related prior knowledge and experiences based on observable evidence and careful reasoning. Indeed, making informed subjective judgments is one of the key features that distinguish a top scientist from a lesser one. Statistics similarly involves subjective judgments, as others have recently argued (Gelman and Hennig 2017).

In the design portion of the Study stage, by definition, information is not yet available from the study being planned. Instead, study design decisions are *preposterior* or *preanalysis* and must be based on (prior) external data and judgment. In this sense, regardless of the subsequent analysis, one could consider the design phase as automatically Bayesian. Designers employ varying degrees of formalism in developing the study design and statistical models. A formal Bayesian approach can be used to either develop a frequentist design (Bayes for frequentist) by, for example, finding a sample size or other design components that ensure

P(power>goal|design &assumptions)>γ,

(see e.g., Shih 1995 for an implementation), or to ensure that Bayesian properties are in an acceptable region (Bayes for Bayes). We recommend practitioners consider additional use of Bayesian approaches, even if only to provide a vehicle for documenting the roles of judgments and as a platform for sensitivity analyses.

All researchers, irrespective of their philosophy or practice, use expert judgment in developing models and interpreting results. We must accept that there is subjectivity in every stage of scientific inquiry, but objectivity is nevertheless the fundamental goal. Therefore, we should base judgments on evidence and careful reasoning, and seek wherever possible to eliminate potential sources of bias.

Science also demands transparency. Ideally, it should be standard practice in all scientific investigations to document *all* subjective judgments that are made, together with the evidence and reasoning for those judgments. Although we recognize that this may be impractical in large studies with many investigators, we believe that it might be facilitated with suitable collaborative working tools. We suggest, therefore, that journals might consider requiring that authors provide such documentation routinely, either in an appendix to a submitted paper or in online supplementary material.

## 4 Case Studies

We present three case studies, each exhibits complexities that require collaborative input from content experts and statisticians. They highlight the roles that expert judgments play and the steps that were taken to ensure that judgments were as objective and scientific as possible.

### 4.1 Expert Judgment in Randomized Clinical Trials

#### 4.1.1 Background on Bayesian Clinical Trials

Bayesian approaches to clinical trial design, conduct and analysis have been shown to offer substantial improvements over traditional approaches in a variety of contexts. See Abrams, Spiegelhalter, and Myles (2004) and Berry et al. (2010), for a range of examples. The Bayesian approach formalizes using prestudy data and expert judgments in design, conduct, and analysis. Importantly, the formalism provides an effective language for discussing interim and final results, for example, by supporting statements such as, “in the light of accruing data, the probability that treatment A is better than treatment B by at least 5 percentage points is 0.95 …” It provides a natural way to compute and communicate predictive assessments such as futility, that is, in the light of current information, what is the probability that, ultimately, the trial will not be definitive. The Block HF and TOXO studies outlined below illustrate many of these characteristics.

#### 4.1.2 The Block HF Study

The BlockHF study (Curtis et al. 2013) provides an example of the benefits of embedding evaluations in the Bayesian formalism. It was an adaptive randomized trial using Bayesian criteria, with specification of all features dependent on collaboration amongst clinical and statistical experts. The abstract states,

We enrolled patients who had indications for pacing with atrioventricular block; New York Heart Association (NYHA) class I, II, or III heart failure; and a left ventricular ejection fraction of 50% or less. Patients received a cardiac-resynchronization pacemaker or implantable cardioverter defibrillator (ICD) (the latter if the patient had an indication for defibrillation therapy) and were randomly assigned to standard right ventricular pacing or biventricular pacing. The primary outcome was the time to death from any cause, an urgent care visit for heart failure that required intravenous therapy, or a 15% or more increase in the left ventricular end-systolic volume index.

Two pacing regimens, biventricular (BiV) versus right ventricular (RiV) were compared using a Cox proportional hazards model. Analyses were stratified by two cardiac devices, with information on the two, stratum-specific hazard ratios (HRs) combined for monitoring. The Bayesian monitoring rules addressed patient safety, stopping for futility, and stopping if the treatment comparison was sufficiently convincing (probability that a clinically meaningful difference was sufficiently high), based on combining evidence over the two stratum-specific HRs. From the statistical analysis section,

An adaptive Bayesian study design allowing up to 1200 patients to undergo randomization was used, featuring sample size re-estimation and two interim analyses with prespecified trial-stopping rules …. An intention-to-treat analysis served as the primary analysis for all outcomes.

The trial resulted in a “win” for biventricular pacing compared to right ventricular, HR 0.74, 95% credible interval (0.60, 0.90). Table 1 presents the monitoring rules which are based on the following,

**Table 1:** θ= a weighted average of stratum-specific log hazard ratios, each comparing BiV versus RiV pacing; PP_0_= posterior probability that the study objective has been met, PRR = posterior probability the study objective has not been met.

Decision boundaries					
	Conclude objective is met and stop study early	Conclude that sample size is sufficient to continue	Determine that sample size is insufficient but elect not to increase sample size	Conclude that sample size must be increased in increments of 175	Stop study for safety
First Interim Analysis	$PP_{0}>0.99$	$0.90\leq PP_{0}\leq 0.99$	*PRR* > 0.9	$PP_{0}<.90$ and PRR≤0.9	P (θ>0|data, prior) ≥ 0.90
Sample Size Re-estimation Phase	N/A	$0.90\leq PP_{0}$	*PRR* > 0.9	$PP_{0}<.90$ and PRR≤0.9	N/A
Second Interim Analysis	$PP_{0}>0.99$	If neither the outcome in column 2 nor the outcome in column 6 occurs, then the study will continue with the current sample size.	P (θ>0|data, prior) ≥ 0.90		

NOTE: See Curtis et al. (2013) for details.

- θ= a weighted average of stratum-specific log HRs, each comparing BiV versus RiV pacing.
- PP_0_ = *P*{*θ* < *log*(0.90)|data} = *pr*{*HR* < 0.90|data}; the posterior probability that the study objective has been met.
- PRR = *P*{*θ* > *log*(0.90)|data} = *pr*{*HR* > 0.90|data}; the posterior probability that the study objective has not been met.
- *P*{*θ* > 0|data} = *pr*{*HR* > 1.00|data}; the posterior probability of a safety concern.

For example, if the probability of meeting the study objective is sufficiently high (PP${}_{0}>0.99$), stop the study; if moderately high ($0.90<{\text{PP}}_{0}<0.99$), continue the study with the current sample size target; if too low (PP${}_{0}<0.90$) but there is a reasonable likelihood of success (PRR < 0.90), increase the sample size. Inclusion of such probability-based rules was an important benefit of using the Bayesian formalism. It supported complex decision rules that communicated effectively with the clinical experts on the monitoring board.

#### 4.1.3 The “TOXO” Trial

When accruing data are consistent with a prior distribution, a trial can be stopped earlier than with traditional, likelihood-based monitoring. However, there are also situations wherein the prior and the data diverge and stopping is delayed, as is the case in the following, post hoc, rerunning of the Community Programs for Clinical Research on AIDS randomized trial of prevention of TOXO conducted in the 1990s. The study compared pharmacological prevention at a subtherapeutic dose with placebo, with all treatment groups carefully monitored for indications of disease. The premise of such prevention studies is that a low dose of a pharmaceutical that is typically used to treat overt disease may also prevent or delay onset. However, even a low dose of a pharmaceutical may induce adverse effects, such as toxicities or resistance to treatment, and consequently, watchful waiting (the placebo “intervention”) may be better than the potentially preventive treatment.

Jacobson et al. (1994) detail the several decisions and expert judgments needed to design and implement the trial, including choice of drugs, inclusion and exclusion criteria, clinical endpoints, and monitoring and analysis plans. The abstract of the article reporting results (Jacobson et al. 1994) states in part,

Pyrimethamine, 25 mg thrice weekly, was evaluated as primary prophylaxis for toxoplasmic encephalitis (TE) in a double-blind, randomized clinical trial in patients with human immunodeficiency virus (HIV) disease, absolute CD4 lymphocyte count of < 200/microL [CD_4_ lymphocytes fight disease; a low level indicates immunodeficiency] (or prior AIDS-defining opportunistic infection), and the presence of serum IgG to Toxoplasma gondii.” …“There was a significantly higher death rate among patients receiving pyrimethamine [compared to control] (relative risk [RR], 2.5; 95% confidence interval [CI], 1.3–4.8; *P* =.006), even after adjusting for factors predictive of survival. The TE event rate was low in both treatment groups (not significant). Only 1 of 218 patients taking [the control intervention] but 7 of 117 taking aerosolized pentamidine for prophylaxis against Pneumocystis carinii pneumonia developed TE (adjusted RR …, 0.16; 95% CI, 0.01–1.79; *P* =.14). Thus, for HIV-infected patients receiving trimethoprim-sulfamethoxazole, additional prophylaxis for TE appears unnecessary.

The Data and Safety Monitoring Board monitored the trial at prespecified, calendar-determined dates using the O’Brien and Fleming (1979) boundaries. Early in the trial, these boundaries require substantial evidence (e.g., a small *p*-value) to stop and make a decision; as the trial approaches the predetermined maximum number of follow-up visits, the criterion is close to that for a fixed sample size trial.

The full, calendar time indexed database was available for the after-the-fact example of how monitoring might have proceeded using a Bayesian approach. This illustrative analysis evaluated the “TOXO or death” endpoint using the Cox (1972) proportional hazards model with adjustment for baseline CD_4_ count. The illustrative trial was stopped when the posterior probability of benefit or the posterior probability of harm became sufficiently high. Importantly, prior elicitation occurred while the trial was being conducted, before any outcome information was available to the elicitees.

#### 4.1.4 Model for the “TOXO” Trial

The HR (relative “TOXO or death” event rate between the two treatment groups) was modeled using a log-linear model with covariates treatment group ($z_{1j}=1$, if participant *j* received pyrimethamine; $z_{1j}=0$, if placebo), and CD_4_ cell count at study entry ($z_{2j}$). The CD_4_ covariate adjusted for possible differences in immune status at baseline between the two groups. Specifically, the log hazard-ratio is,

$$\text{log}\;(\text{hazard}\;\text{ratio})=\beta_{1}z_{1j}+\beta_{2}z_{2j}$$

with $\beta_{1}<0$ indicating a benefit for pyrimethamine. A flat prior was used for the CD_4_ effect (*β*_2_), and a variety of priors were developed for the pyrimethamine effect (*β*_1_). The choice of the Cox (1972) model and the use of a noninformative prior for *β*_2_ were judgments of the statisticians. Though the Cox model has become the default choice when modeling the time to an event, it is based on important assumptions, namely that the hazard functions for both interventions are proportional and censoring is noninformative. As such, the model should only be adopted after careful consideration by experts, such as in this example. The choice of a noninformative prior distribution for *β*_2_ reflects the clinicians’ and statisticians’ judgments that no information was available in advance of the trial on the association of CD_4_ with TOXO incidence.

#### 4.1.5 Elicitation in the “TOXO” Trial

As described in Carlin et al. (1993) and Chaloner et al. (1993), prior distributions for *β*_1_ were elicited from five content experts—three HIV/AIDS clinicians, one person with AIDS conducting clinical research, and one AIDS epidemiologist. Elicitation occurred while the trial was in progress, only pretrial information was available to the elicitees. Two additional priors were included in the monitoring—an equally weighted mixture of the five elicited priors and a noninformative flat prior (equivalent to using the normalized, partial likelihood as the posterior distribution).

Elicitation targeted potentially observable, clinically meaningful features and then transformed responses to parameters of the Cox model. That is, rather than directly elicit a prior for the hazard ratio, each elicitee was asked to report their best estimate of the probability of TOXO or death in two years under placebo $\left(P_{0}\right)$, then draw a picture of the distribution of the two-year probability under pyrimethamine, conditional on the estimate under placebo ($\left[P_{\text{pyri}}|P_{0}\right]$). Then, for each elicitee these conditional distributions were converted to a prior distribution for the log(hazard ratio) using the transformation, $\beta_{1}=\;\text{log}\;(1-P_{0})-\;\text{log}\;(1-P_{\text{pyri}})$.

**Fig. 2:**
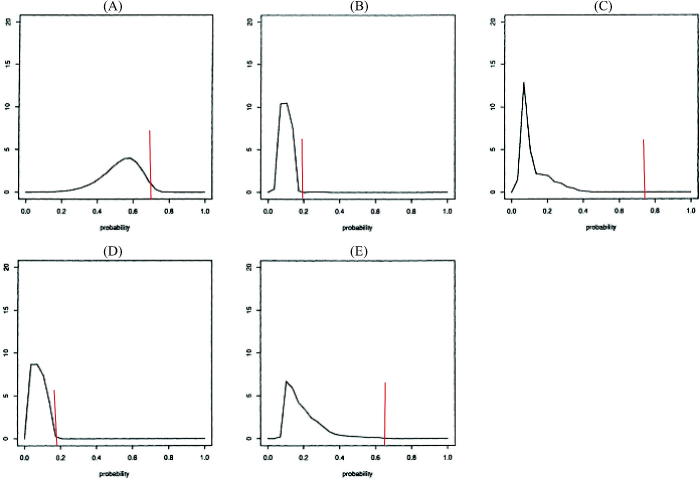
Elicited priors for the five elicitees. The red vertical line at *P*_0_ is the “best guess” two-year incidence of TOXO or death under placebo. The smoothed densities are for two-year TOXO or death incidence under pyrimethamine, conditional on the placebo rate.

Figure 2 displays the elicitation results. At trial initiation, there was little known about the baseline incidence of TOXO, and so the content experts based their distributions on general expertise and analogy with other endpoints and contexts. The range of the five reported two-year incidence probabilities under placebo was wide, but elicitees believed that TOXO had a non-negligible baseline incidence. All elicitees were optimistic regarding pyrimethamine’s benefit, with experts C and E the most optimistic, placing all of their probability distribution for incidence under pyrimethamine way below their estimate of incidence under placebo.

While a degree of optimism may be needed to motivate conducting a trial, ethics require that there be sufficient equipoise (uncertainty) regarding which treatment is superior in order to initiate a trial. The elicited priors in this example probably express too strong a belief in the efficacy of pyrimethamine to be used in monitoring an actual trial, but using them in this illustrative monitoring exercise with comparison to likelihood-based monitoring, effectively highlights issues we discuss in Section 4.1.7.

#### 4.1.6 Monitoring Results for the “TOXO” Trial

The actual trial was monitored at calendar dates (15 Jan 1991, 31 Jul 1991, 31 Dec 1991, 30 Mar 1992). At the December 31, 1991 meeting, the monitoring board recommended stopping the trial for futility, because the pyrimethamine group had not shown significantly fewer events, and the low overall event rate made a statistically significant difference in efficacy unlikely to emerge. Additionally, an increase in the number of deaths in the pyrimethamine group relative to the placebo indicated a safety issue.

**Fig. 3:**
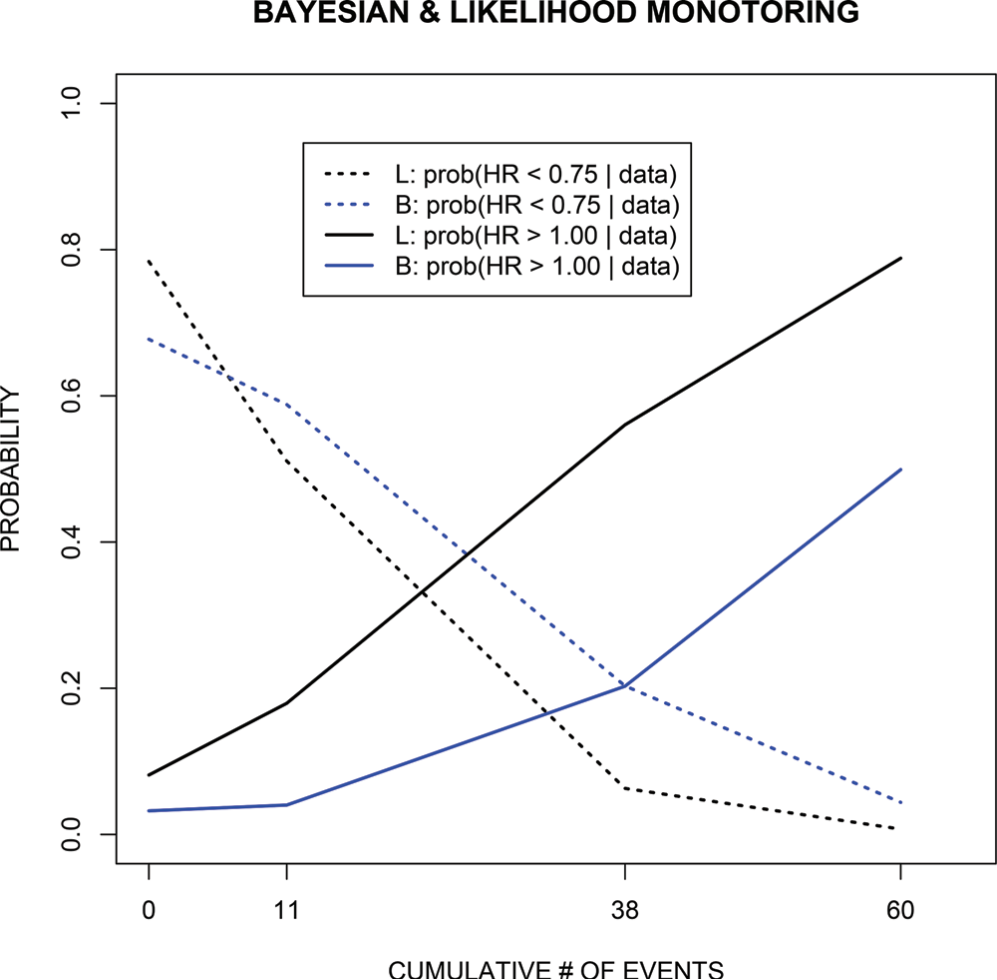
Posterior probability of benefit (hazard ratio ≤0.75) and harm (hazard ratio > 1.0) for the mixture prior (blue lines) and for monitoring based on the partial likelihood (black lines), which is equivalent to using a flat (improper) prior. Monitoring was at calendar dates (15 Jan 1991, 31 Jul 1991, 31 Dec 1991, 30 Mar 1992) with the X-axis indicating the cumulative number of toxoplasmosis or death events from the combined arms.

For the illustrative, after the fact monitoring example, Figure 3 displays posterior probabilities of benefit (HR ≤0.75, equivalently, $\beta_{1}\leq \;\text{log}\;(0.75)$ in the Cox model), and harm (HR $>1.0;\beta_{1}>0$) for an equally weighted mixture of the five elicited prior distributions (denoted by “B”), and for a flat prior that generates partial likelihood-based/traditional monitoring (denoted by “L”). For example, $\beta_{1}=\;\text{log}\;(0.75)$ indicates that the hazard rate (event rate) in the pyrimethamine group is 75% of that in the control group; $\beta_{1}=0=\;\text{log}\;(1.0)$ indicates equal rates.

As displayed in Figure 2, elicitees believed that TOXO had a nonnegligible incidence and that pyrimethamine would have a substantial prophylactic effect. Thus, each prior and their mixture are to varying degrees far from the accruing information in the trial. Consequently, monitoring based on the partial likelihood, which can be considered “flat prior Bayes,” gives an earlier warning of harm compared to monitoring based on the mixture prior. The mixture required considerably more information to overcome the a priori optimism of the elicitees. Also, the posterior probability of harm based on monitoring with any single prior in Figure 2 would lag behind that based on the partial likelihood, with use of prior A, B, or D giving an earlier warning than use of priors C or E.

#### 4.1.7 Discussion of the “TOXO” Trial

Traditional, non-Bayesian monitoring depends on several judgments. For example, trial design including maximum sample size depends on assumptions about baseline event rates, the HR, principal efficacy and safety endpoints, the statistical model, etc. In the Bayesian approach, a subset of these features are given prior distributions, in the TOXO example by independent priors for the slopes on the log HR and on the baseline CD_4_ value. These priors, along with other design features determine the monitoring frequency, the shape of monitoring boundaries (flat, increasing, decreasing) and associated values. Boundaries are necessary for all monitored trials, determining them can be either Bayesian or frequentist.

The after-the-fact analysis highlights ethical issues generated by real-time use of expert knowledge. It shows that if the elicited priors had been used in the actual clinical trial monitoring, then due to their optimism it is very likely that trial stopping and other decisions would differ from those produced by likelihood-based (flat prior) monitoring. However, in early phase clinical applications, and in nonclinical applications, use of such optimistic priors can be appropriate and effective.

In the example, stopping would have been delayed, highlighting the question of whether it would be ethical to continue beyond a traditional stopping point due to prior beliefs that pyrimethamine would have a strong, prophylactic effect. Of course, if pyrimethamine had performed well, stopping would have been earlier than that based on traditional methods, also raising an ethical issue for some. And, if the priors had been pessimistic, but the data moderately optimistic, the Bayesian analysis would have tempered enthusiasm (delayed stopping) until convincing, positive, data had accrued.

The TOXO example shows one possible effect of using prior distributions in clinical evaluations designed to be definitive (Phase III). The example is based on the mixture that equally weighted the five prior distributions. The posterior distribution is also a mixture, but the posterior weights give more influence to the priors that are most compatible with the data, so there is some degree of automatic adjustment. Other options include separately monitoring with each prior and then at each monitoring point determine stopping based on the “majority rule” or requiring unanimity. Each choice will produce its own operating characteristic, so extensive simulations are needed to understand properties.

To summarize, in clinical trial monitoring prior information can have two main effects:

1. If prior information conflicts with the available data at the time of monitoring, it may suggest continuing the trial, at least until the next review. This situation can arise when the experts are more optimistic than the emerging data, as in the TOXO trial, or when they are more pessimistic. In either case, continuing the trial long enough to obtain sufficient evidence to convince the content experts may have an added benefit that the results would translate to practice relatively rapidly.
2. If prior information supports the available data at the time of monitoring, it may suggest terminating early, on the grounds that sufficient evidence now exists to make a decision.

An increasing number of trials are utilizing prior information (Abrams, Spiegelhalter, and Myles 2004; Berry et al. 2010), and the advent of such trials emphasizes the importance of prior judgments being made as carefully and as objectively as possible. Judgments should be sought from a sufficient number of content experts to properly represent the range of views. Determining the number and type of elicitees is, itself, an exercise in study design. Of course, prior distributions should be elicited carefully and rigorously, as described, for instance, in O’Hagan (2018).

### 4.2 Expert Judgment in Environmental Modeling

#### 4.2.1 Background: UK Carbon Flux

This case study describes the estimation of a parameter in a complex environmental modeling problem. As a party to the Kyoto protocol, the United Kingdom is committed to specific target reductions in net emissions of greenhouse gases. Monitoring progress toward these targets is challenging, involving accounting for numerous sources of emissions. One potential mitigating factor is the ability of vegetation to remove carbon dioxide from the atmosphere, thereby acting as a “carbon sink.” However, accounting for this is also extremely complex. During the day, through the process of photosynthesis, vegetation absorbs carbon dioxide and releases oxygen, using the carbon to build plant material. Photosynthesis requires sunlight, chlorophyll in green leaves and water and minerals gathered by the plant’s roots, so the efficiency of carbon removal depends on factors such as weather, leaf surface area, and soil conditions. Conversely, carbon is released at night, carbon extracted from the atmosphere and converted to biomass will eventually be released as the plant ages and dies, and carbon in leaf litter is released by microbial action in the soil. The Sheffield Dynamic Global Vegetation Model (SDGVM) was built with mathematical descriptions of all these processes to predict net carbon sequestration due to vegetation (Woodward and Lomas 2004). For a given site, the model takes many inputs describing the type of vegetation cover and soil at the site, together with weather data, to estimate Net Biosphere Production (NBP), that is, the net decrease in atmospheric CO_2_, from that site over a given time period.

This case study used SDGVM to estimate the total NBP for England and Wales in the year 2000. It is important to recognize that there is inevitably uncertainty about all the model inputs. Uncertainty about inputs induces uncertainty about model outputs, and the study sought to quantify the output uncertainty in the form of a probability distribution for the total NBP. Details are reported in Kennedy et al. (2008) and Harris, O’Hagan, and Quegan (2010).

Before considering the statistical aspects of this case study, we first note that considerable content expertise had already gone into the development of SDGVM. Based on the available scientific knowledge, expert judgments were made in choosing the structure of the model and the equations that describe each of its biological processes. Care went into these choices to ensure that they were reasonable and scientifically defensible. However, in such modeling, it is usually unrealistic to include every process to the greatest level of detail and complexity that is believed to be applicable. First, the more complex and detailed the model, the longer it will take to compute; for practical reasons, it may be necessary to compromise on complexity. Second, more complex models may be less robust and reliable in their predictions, because at the highest level of detail, there is often less scientific consensus about the equations and the parameters within them. In addition, models with a large number of parameters may suffer from overfitting (Hawkins 2004). Judgments about the optimal degree of complexity to achieve a computable, accurate, and reliable representation of the phenomenon being modeled often demand a particularly high level of expertise.

#### 4.2.2 Input Distributions for the SDGVM

The model was run over 707 grid cells covering England and Wales. For each grid cell, given the land cover in that cell, and given input parameters describing the soil composition and properties of the vegetation types, the model was first “spun-up” for 600 years. That is, the model was initialized with a default set of inputs describing the state of the vegetation in terms of ages, heights, leaf density, etc., and the state of the soil in terms of moisture content, age and quantity of organic matter, etc. Then the model was run forward for 600 years using historic climate data at that site from 1400 to 2000, to stabilize the vegetation and soil at states representative of how that site would have been in 2000. The model was then run forward for one more year using weather data from 2000, and the NBP for the year was computed for each grid cell. The NBP for England and Wales in 2000 is the sum of the NBP values across all the grid cells.

Care was taken to quantify the uncertainty in the various inputs as described previously (O’Hagan 2012). Briefly, details are described below.

- *Soil composition*: A publicly available soil map for England and Wales (Bradley et al. 2005) was used to provide estimates of the soil composition in each grid cell. Because the map was created at a higher resolution than the grid cells used for SDGVM, figures were averaged over each grid cell to provide an estimate. The variance of the same figures over a grid cell, divided by the number of map points in the cell, was used to quantify uncertainty around the estimates for a grid cell. However, the variance was increased by a factor to represent (a) additional uncertainty in the map data and (b) spatial correlation within the cell. The decision to use an increased estimate of uncertainty in the model was based on an expert judgment on the part of the statisticians, in consultation with content experts.
- *Land cover*: A map of land cover was also publicly available (Haines-Young et al. 2000), derived from satellite observation. The original analysis reported in Kennedy et al. (2008) did not quantify uncertainty in land cover. However, unlike the soil map, the content experts felt that the uncertainties in land cover were large and there could be biases in the process by which land cover is inferred from the satellite readings. In a subsequent analysis (Cripps, O’Hagan, and Quaife 2013; Harris, O’Hagan, and Quegan 2010), a statistical model was built to quantify uncertainty in land cover maps derived from remote sensing. The analysis of England and Wales NBP in 2000 was then extended to account for the additional uncertainty. The method makes use of the “confusion matrix,” which for the Haines-Young et al. (2000) map was given by Fuller et al. (2002). The confusion matrix is a contingency table based on a large survey of actual, ground-truth, land cover, and shows, for each ground-truth vegetation type, the frequency with which it was classified by the Haines-Young et al. (2000) map in each vegetation type. The statistical analysis required probabilistic inversion of the confusion matrix to derive, conditional on the satellite land cover, the probabilities of the various ground-truth cover. The careful expert judgments of statisticians and content experts are delineated in Cripps, O’Hagan, and Quaife (2013).
- *Vegetation properties*: SDGVM classifies land cover into plant functional types (PFT). For England and Wales, we used four PFTs—evergreen needleleaf trees, deciduous broadleaf trees, crops, and grassland. Each PFT is associated with a set of quantities, including maximum age, stem growth rate and leaf lifespan. However, most of these inputs were missing. Some properties had been estimated experimentally, but only for very few individual species within a given PFT. We therefore used expert elicitation to construct a probability distribution for each parameter. Elicitation is an area where it is particularly important to take care to avoid biases that commonly arise in subjective judgments of probabilities (Kynn 2008). Another article arising from the Symposium addresses issues related to elicitation in detail (O’Hagan 2018).
- *Weather*: Weather data, such as temperature, precipitation, and cloud cover, were available for each grid cell for every day in 2000. There are no doubt errors in these data, due not only to errors in the underlying daily measurements, but also to the fact that those measurements have been interpolated to produce the data at the level of grid cells. Nevertheless, it was felt that uncertainty in these inputs was relatively small and could not be quantified without adding extra assumptions. The decision not to trade (potentially unreasonable) assumptions for (potentially increased) precision for weather data was a judgment made jointly by statisticians and content experts.

#### 4.2.3 Propagating the Input Uncertainty in the SDGVM

To propagate input uncertainty through a mechanistic model such as SDGVM, the simplest and most direct approach is by Monte Carlo. A large sample of random draws are made from the probability distributions of the inputs, and the model is run for each sampled input set. The resulting set of outputs is then a random sample from the output uncertainty distribution. However, like many models that are built to describe complex physical processes, the computational load in running the SDGVM model is substantial, and it would not have been feasible to propagate parameter uncertainty through the model in this way at even one of the grid cells. The problem of quantifying uncertainty in complex computer models has acquired the name uncertainty quantification (UQ), and various tools are available to enable uncertainty propagation. The analysis used Gaussian process emulation (O’Hagan 2006; Oakley 2016), which is probably the most popular UQ technique. Even so, it would not have been feasible to build emulators at every one of the 707 grid cells; 33 were chosen by content experts to represent, in their expert judgment, the range of conditions across England and Wales, and GP techniques were adapted to infer the magnitudes of output uncertainty at the other 674 cells. The computational techniques and accompanying statistical theory to manage the analysis are set out in Kennedy et al. (2008) and Gosling and O’Hagan (2006).

#### 4.2.4 Results of the UK Carbon Flux Study

The content experts were certainly interested in knowing how much uncertainty in the total NBP would be induced by uncertainty in the inputs. Their instinctive approach to estimating the total NBP was to run the model just once with all of the inputs set to their expected values; we call their idea the plug-in estimate. Yet, the NBP output from SDGVM is a highly nonlinear function of its inputs. Therefore, the expected value of NBP, when we allow for uncertainty in the inputs, is not equal to the plug-in estimate. Statisticians incorporated the input uncertainty and computed the expected NBP for England and Wales in 2000 as 7.46 Mt C (megatonnes of carbon). By contrast, the plug-in estimate was 9.06 Mt C. Not only is this a substantial difference, but the standard deviation was estimated as 0.55 Mt C. Therefore, the total NBP was probably in the interval (6.36 Mt, 8.56 Mt C) and very likely to be less than the plug-in estimate.

The result was very surprising for the content experts. It seems that the explanation lies in their estimates of vegetation properties. In effect, the experts had estimated values that were approximately optimal for the plants to grow and absorb CO_2_. Any deviation from these values led to lower NBP. For the total NBP to be even close to the plug-in estimate required all the parameter values to be close to their estimates, a joint event that had very low probability.

In this case study, the combination of expert judgments from both content experts and statisticians, applied with as much care, rigor, transparency, and objectivity as possible, led to a scientific result that certainly highlighted the role of expert judgment and the statistical quantification of uncertainty, and also prompted new questions regarding the accuracy of current methods for carbon budgeting, with important implications for the science of global climate change. For example, see Cripps, O’Hagan, and Quaife (2013). The cycle of scientific investigation was thereby renewed.

## 5 Conclusions

Expert scientific judgment involves carefully considered conclusions and decisions based on deep knowledge and skills. The use of expert judgment is essential in and permeates all phases of scientific and policy studies. Expert knowledge is information; to ignore it or fail to obtain it incurs considerable opportunity costs. Judgments should be as objective as possible and based on data when available. Anything less is unscientific. Yet, deciding what data are relevant always involves degrees of judgment.

Written documentation that logs decisions is critical for informing stakeholders of how and in which study components (design, conduct, analysis, reporting, and decision-making) the principal judgments had impact. By “impact,” we mean that such judgments led to decisions that directly affected (or had potential to alter or influence) any aspect of how the study was conducted and/or results interpreted.

Elicitation of expert judgments to produce probability distributions that represent uncertainty about model parameters can be conducted informally, but such judgments are easily affected by unconscious cognitive biases, such as overoptimism or failure to recall all relevant evidence. When such distributions form an important part of a scientific activity, the expert judgments should be elicited scientifically and as objectively as possible, minimizing relevant sources of bias. Doing so requires a carefully designed process, an elicitation protocol, as fully discussed by O’Hagan (2018).

It should be unsurprising that statisticians have essential roles as scientists, ideally serving as leaders or coleaders in all study aspects. Indeed, virtually all aspects of a study have statistical content, though almost no aspects are solely statistical. Consequently, we advise statisticians to more pro-actively promote the needs for statistical principles to permeate all stages of research studies. Furthermore, we advise research authorities, such as journal editors and funding agencies, to recommend or even require thorough collaboration with one or more experts in statistics throughout the duration of all projects.

The 2017 Symposium will not produce a single position document on statistical practice like the “The ASA Statement on *p*-Values” that resulted from the 2015 ASA Board meeting (Wasserstein and Lazar 2016). However, we echo the call in Gibson (2017) for statisticians to better advocate for the importance of their involvement throughout the scientific process. Finally, we applaud stakeholders, such as the National Institutes of Health (Collins and Tabak 2014) and the American Association for the Advancement of Science (McNutt 2014) in the USA, and National Institute for Health and Care Excellence (NICE 2012) and HM Treasury (2015) in the UK for leading calls for increased statistical rigor and understanding of uncertainty. We encourage additional funding agencies, journals, hiring and promotion committees, and others to join in the call for higher scientific standards, statistical and otherwise. Science in the twenty-first century and beyond deserves nothing less.

## Supplementary Material

Supplemental Material
