# Supplementary figures and images for: The Role of Expert Judgment in Statistical Inference and Evidence-Based Decision-Making
Source: Am Stat. 2019 Mar 20;73(0 1):56–68. doi: 10.1080/00031305.2018.1529623 (PMC6474725; doi:10.1080/00031305.2018.1529623)

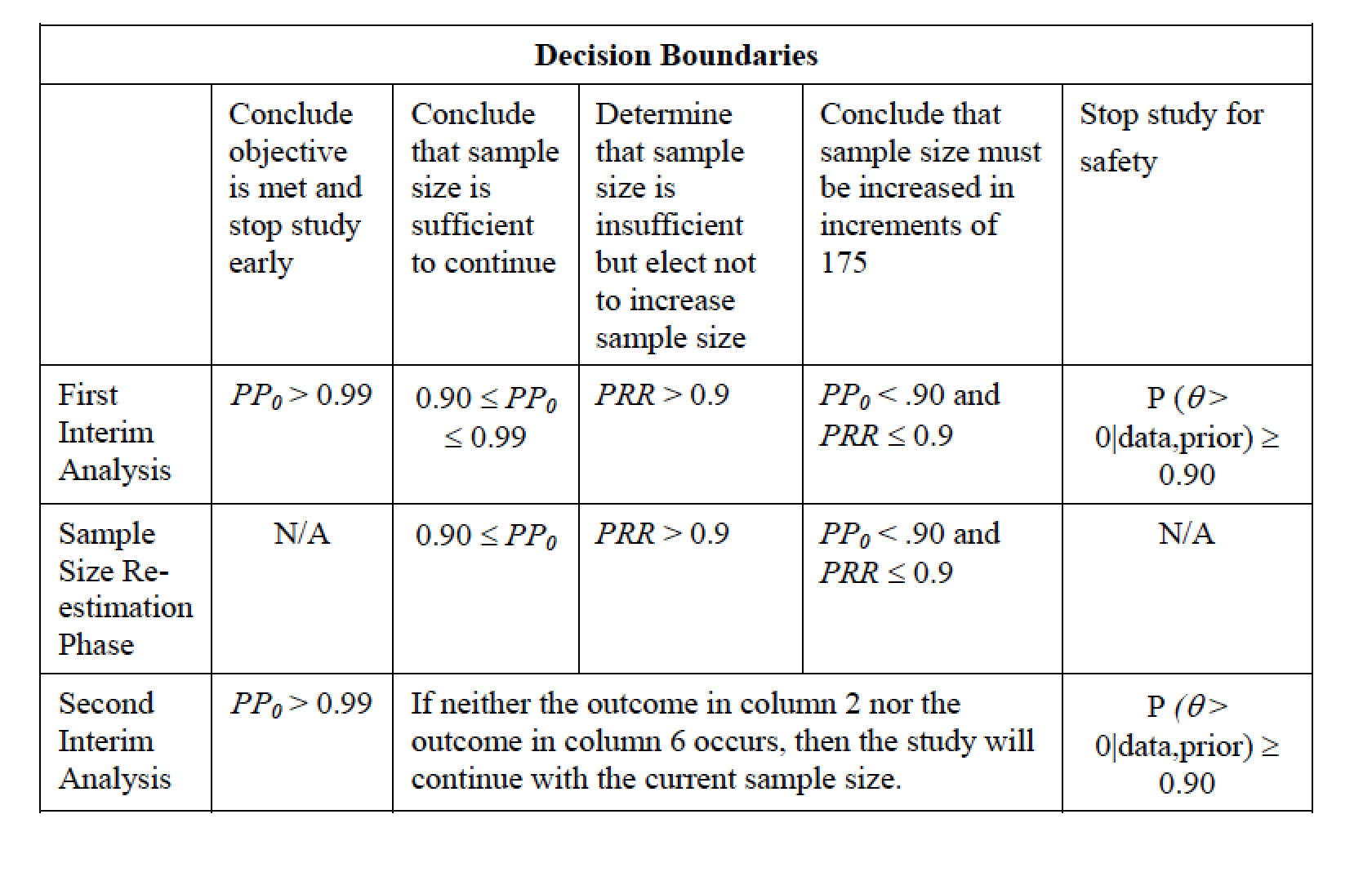

Supplement: Supplemental Material [file UTAS_A_1529623_SM1519.zip › UTAS_A_1529623/blockHF-rules.png]

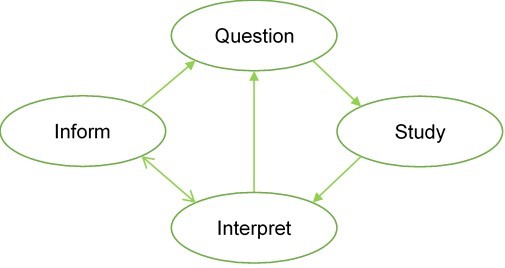

Supplement: Supplemental Material [file UTAS_A_1529623_SM1519.zip › UTAS_A_1529623/Fig1.jpg]

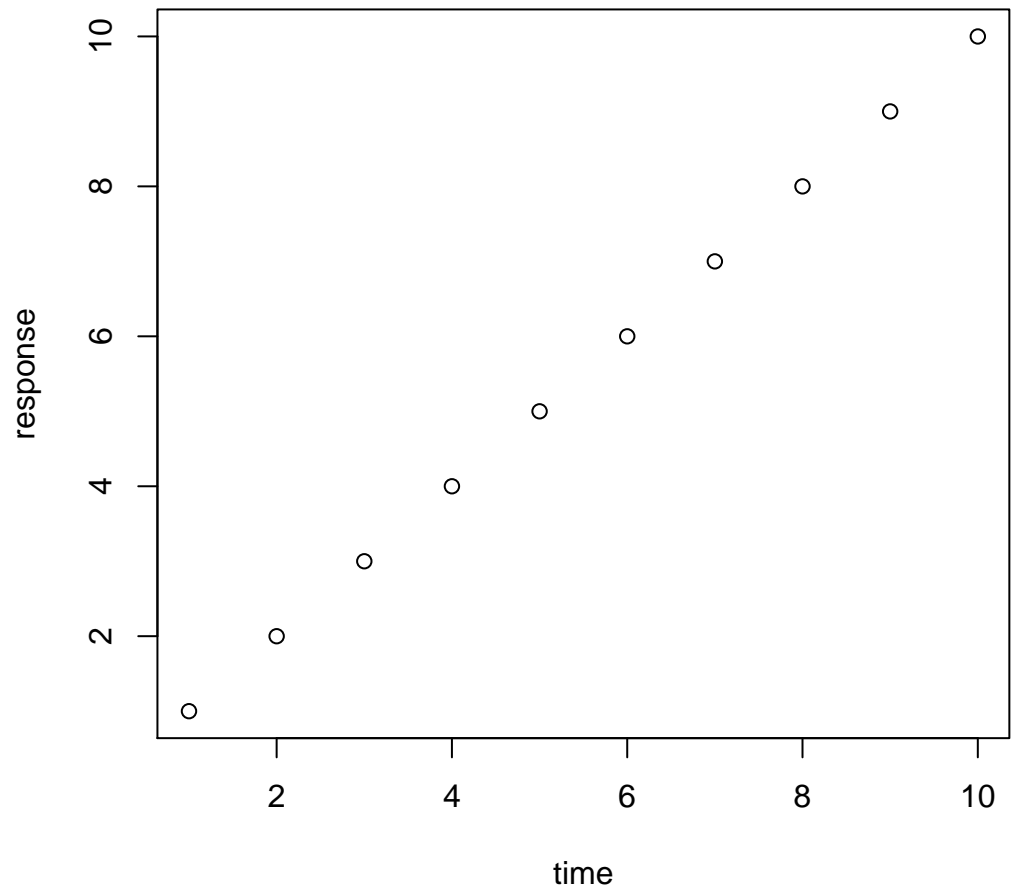

Supplement: Supplemental Material [file UTAS_A_1529623_SM1519.zip › UTAS_A_1529623/fig1.pdf]

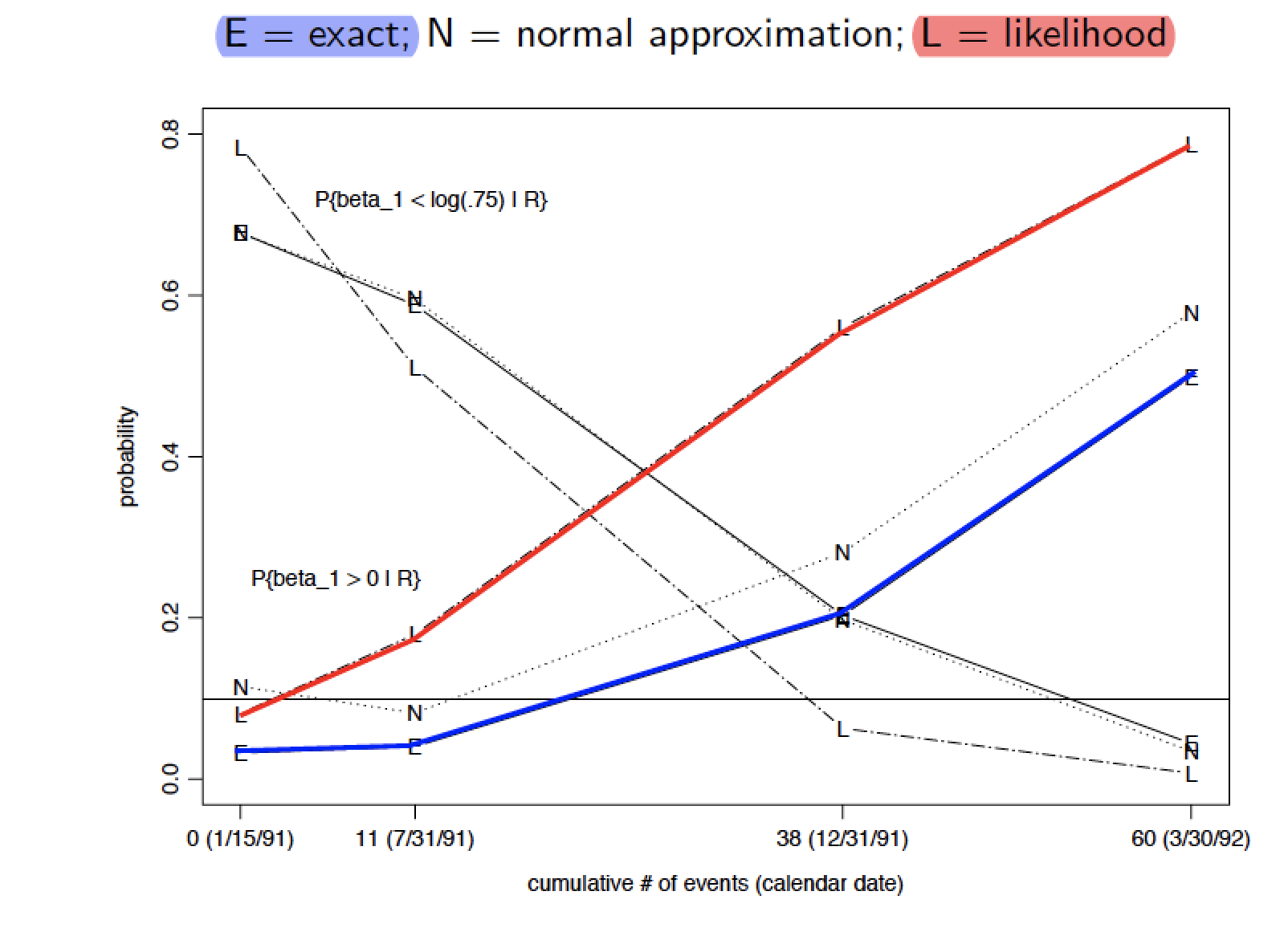

Supplement: Supplemental Material [file UTAS_A_1529623_SM1519.zip › UTAS_A_1529623/Fplot2Highlighted.png]

# BAYESIAN & LIKELIHOOD MONOTORING

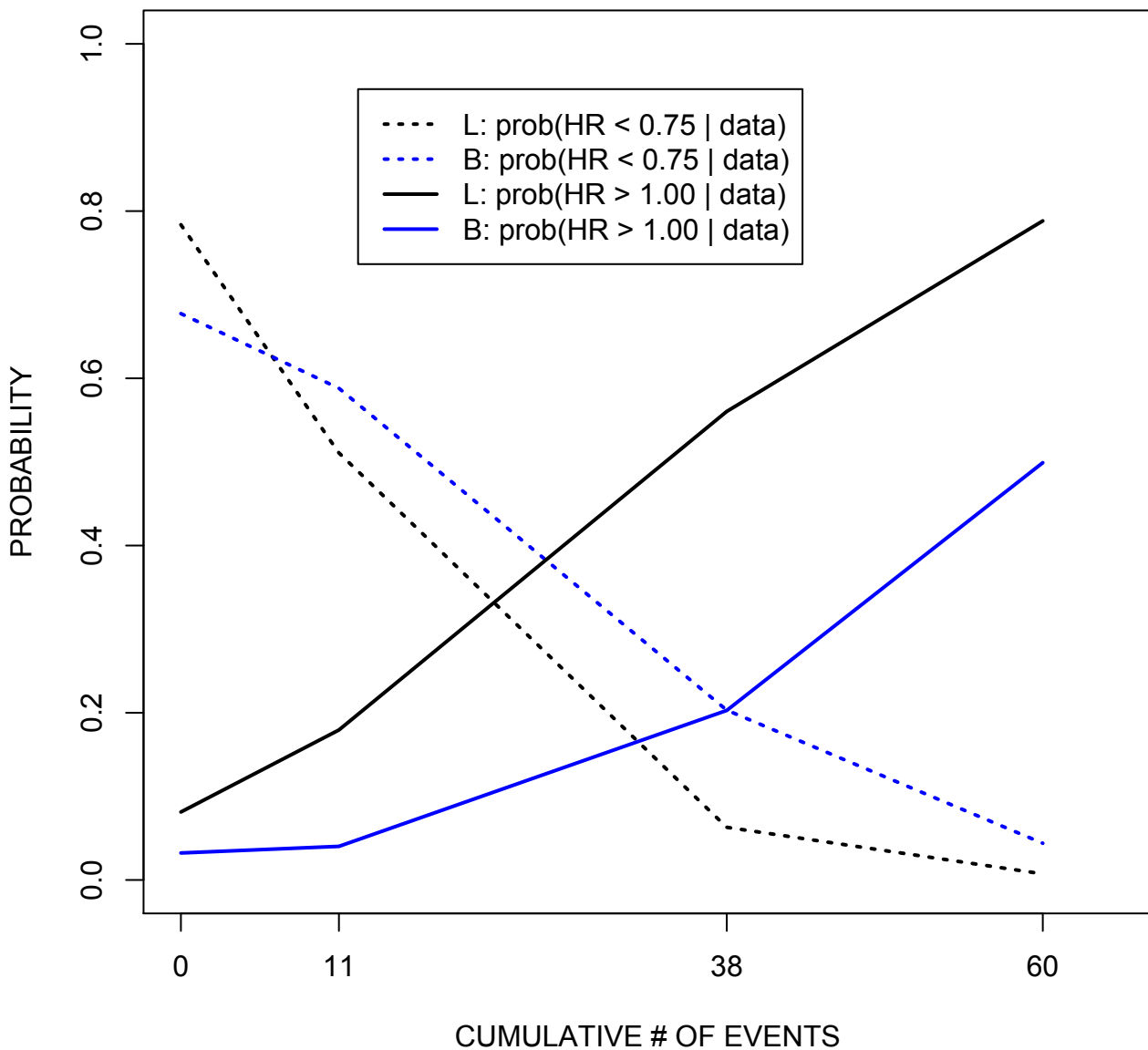

Supplement: Supplemental Material [file UTAS_A_1529623_SM1519.zip › UTAS_A_1529623/toxomonitoringPlot.pdf]

A

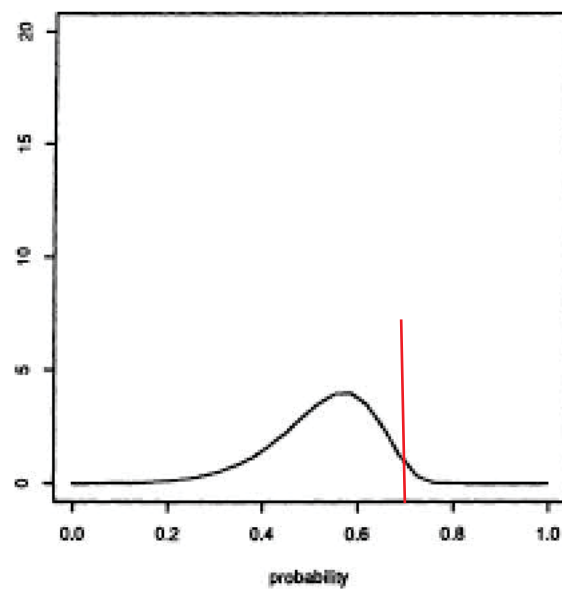

B

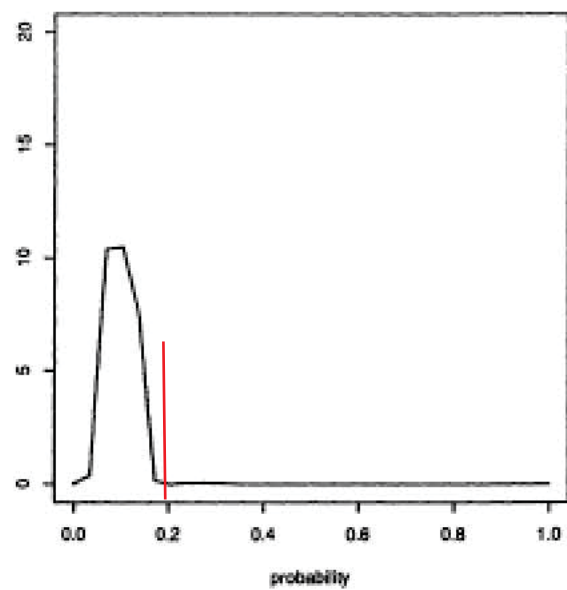

C

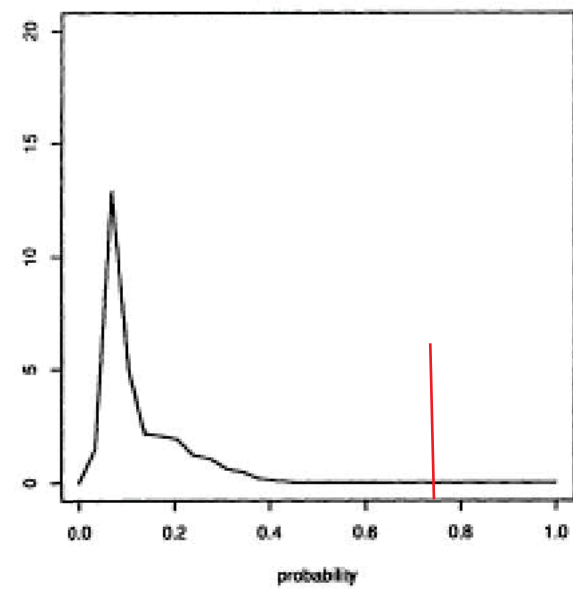

D

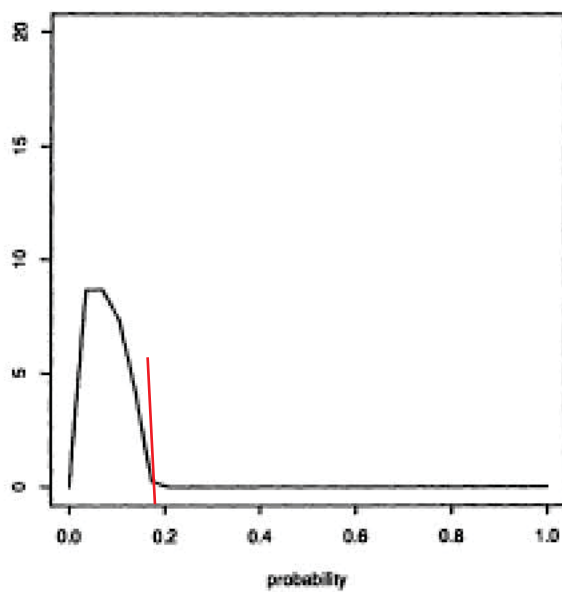

E

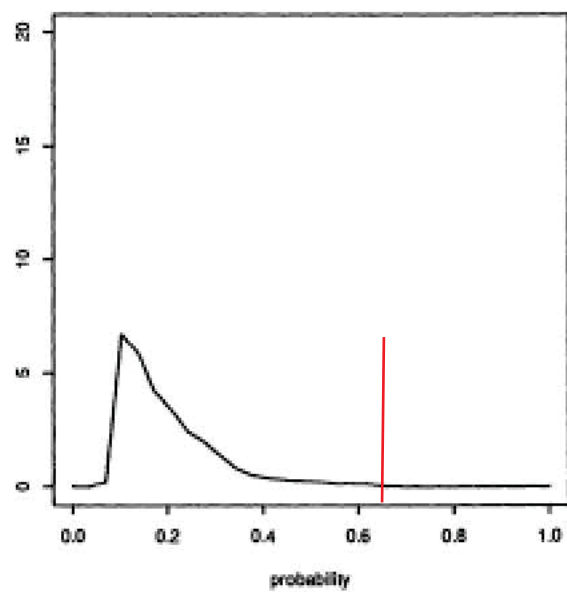

Supplement: Supplemental Material [file UTAS_A_1529623_SM1519.zip › UTAS_A_1529623/toxoPriors.pdf]
